# The relationship between serum MOG-IgG titer status and titers and the prognosis of pediatric MOGAD: a retrospective cohort study

**DOI:** 10.3389/fped.2026.1767553

**Published:** 2026-04-09

**Authors:** Mengfan Pei, Yuyang He, Shan Huang, Cong Yao, Jing Chen, Lanhong Xiang, Chunhui Yuan, Hongmin Zhu

**Affiliations:** 1 Department of Rehabilitation Medicine, Wuhan Children's Hospital Graduate Joint Training Base, School of Medicine, Wuhan University of Science and Technology, Wuhan, China; 2 Department of Rehabilitation Medicine, Wuhan Children’s Hospital, Tongji Medical College, Huazhong University of Science and Technology, Wuhan, China; 3 Health Care Department, Wuhan Children’s Hospital, Tongji Medical College, Huazhong University of Science and Technology, Wuhan, China; 4 Department of Laboratory Medicine, Wuhan Children’s Hospital, Tongji Medical College, Huazhong University of Science and Technology, Wuhan, China

**Keywords:** child, clinical characteristics, myelin oligodendrocyte glycoprotein, myelin oligodendrocyte glycoprotein associated disease, prognosis

## Abstract

**Background:**

To explore the clinical value of MOG-IgG serum titer change and duration in predicting the relapse of MOGAD in children.

**Methods:**

The clinical data, imaging data and laboratory examination results of 63 children with MOGAD were analyzed, and the relationship between serum MOG-IgG titer, titer status and disease relapse was examined.

**Results:**

(1) 55 children were positive for MOG-IgG serum titers. According to the titer status, there were 24 cases in the transient positive group and 31 cases in the continuous positive group. There were 36 cases in the low MOG-IgG titer group (≤1:32) and 19 cases in the high MOG-IgG titer group (>1:32). Eight cases were children with overlap syndrome. (2) Compared with the transient positive group, the probability of relapsing in the persistent positive group may be higher (*χ*2 = 4.685, *P* = 0.030), and the number of relapses may be higher (Z = 2.254, *P* = 0.024). Persistent positive MOG-IgG titers showed a poor long-term prognosis (Z = 2.490, *P* = 0.013). The time to negative MOG-IgG titers in the monophasic course group was shorter than that in the relapsing group (*P* < 0.05). Compared with the low titer group, the high titer group had more cerebrospinal fluid cells (Z = 2.100, *P* = 0.036), and the high titer group had a longer duration of glucocorticoid use (Z = 3.088, *P* = 0.002). (3) In multivariate logistic regression, the time of glucocorticoid use was an independent influencing factor of serum MOG-IgG titer at onset (OR: 1.483, 95% CI = 1.105–1.991, *P* = 0.009), and the EDSS score (prognostic score) at the third year was an independent influencing factor of MOG-IgG titer status (OR: 3.021, 95% CI = 1.030–8.864, *P* = 0.044). (4) The antibody titer in the monophasic group showed a gradual negative trend, and the titer in the relapsing group fluctuated.

**Conclusions:**

The persistent positive state of MOG-IgG is an important marker of recurrence and poor prognosis. Children with high titers of MOG-IgG at onset or those who do not turn negative within 3 months may be considered to extend the immunotherapy period. Careful examination of the serum titer status and titer of MOG-IgG, combined with clinical data, can guide the clinical further analysis of the prognosis of MOGAD.

## Introduction

Myelin oligodendrocyte glycoprotein antibody-associated disease (MOGAD) is an immune-mediated inflammatory demyelinating disorder of the central nervous system, distinguished by its pathological features from multiple sclerosis and aquaporin-4 antibody-positive neuromyelitis optica spectrum disorders (1). The disease can affect both children and adults, with a relatively higher incidence rate in children (2). MOGAD has diverse clinical phenotypes, including optic neuritis, myelitis, brainstem syndrome, and encephalitis (3). Although some patients respond well to acute phase treatment, MOGAD has a high tendency for relapse and disability, which may lead to long-term neurological deficits such as visual and motor impairments (4). It seriously affects the quality of life of patients, so it is crucial to identify the risk factors associated with relapse and prognosis.

The course of MOGAD exhibits significant heterogeneity, manifested as a monophasic or relapsing course (4). Multiple factors are associated with the risk of relapse, including gender (5), race (6), history of immune diseases (7), cerebrospinal fluid protein (8) and increased number of cells (7). In addition, not receiving maintenance treatment after the first onset may also be associated with relapse (8). Therefore, early identification of risk factors associated with relapse and poor prognosis is crucial for guiding individualized treatment in clinical practice.

The role of changes in serum MOG-IgG titers and their persistent positive status in predicting relapse and prognosis is currently a research focus. Research has shown that higher antibody titers or persistent positivity may be associated with an increased risk of relapse, while a decrease in titers or a negative result may indicate a lower risk of relapse (9). However, existing conclusions are controversial, with some studies showing no clear association between antibody titers and MOGAD progression (10). The titers of MOG-IgG should not be used as the only basis for relapse and long-term immunoregulatory treatment (11). These controversies may stem from differences in research design, patient populations, testing methods, and titer cutoff values. Therefore, it is necessary to study unified detection standards and clarify the predictive value of MOG-IgG titers and their dynamic changes in different clinical subtypes and populations, in order to resolve the current controversies.

This study conducted a retrospective analysis to explore the clinical characteristics, serum MOG-IgG titers, and the relationship between relapse and prognosis in children with MOGAD. The focus was on evaluating factors such as the Expanded Disability Status Scale Score (EDSS), past episodes, and duration of initial glucocorticoids therapy, in order to provide evidence-based management for individualized clinical practice.

## Materials and methods

### Study design and population

This study included 63 children diagnosed with MOGAD who were hospitalized at Wuhan Children's Hospital affiliated with Tongji Medical College of Huazhong University of Science and Technology (2014.10-2024.3). Retrospective statistical analysis was performed on clinical numbers, gender, age, pre-infection, nature of disease, frequency, time of disease, subtype, head MRI at the beginning and follow-up, serum MOG-IgG titer level, time of negative MOG-IgG transition, and use of glucocorticoids.

The inclusion criteria are: (1) onset age ≤ 18 years old, the patient was previously healthy; (2) All participants underwent comprehensive clinical evaluation; (3) The serum CBA method detected MOG-IgG as positive; (4) Clinical manifestations include one or a combination of the following: optic neuritis, myelitis, encephalitis or meningoencephalitis, brainstem encephalitis; (5) MRI results related to CNS demyelination; (6) Exclude other diagnoses. Exclusion criteria: Patients with other autoimmune diseases (such as Sjogren's syndrome, systemic lupus erythematosus, etc.) or those who cannot receive MRI scans.

Clinical relapse is defined as: A relapse was defined as any new CNS sign or symptom lasting at least 24 h, supported by clinical examination or radiologic findings, and occurring at least 1 month after a prior attack, which is consistent with the international MOGAD relapse definition (2, 12). Use EDSS scoring to record disability outcomes, selecting scores obtained 3 years after onset (the minimum follow-up period of the cohort). EDSS mainly evaluates the severity of neurological dysfunction and disease in children, with a scoring range of 0–10 points. The higher the score, the more severe the neurological deficit (12).

### MOG antibody status and cerebrospinal fluid studies

Blood samples from the patient were sent to Wuhan Kangsheng Global Laboratory for detection of autoimmune antibodies (such as AQP-4-IgG, MOG-IgG, GFAP-IgG, and NMDAR-IgG) using the live-CBA method. This study mainly included information on the selection of serum MOG-IgG, GFAP-IgG, and NMDAR-IgG. The positive status of MOG-IgG is determined as a serum MOG-IgG titer ≥ 1:10. The initial follow-up time for MOG-IgG serum titer is 3 months after the onset of the disease, and follow-up is conducted every 6 months thereafter. If the titer turns negative during follow-up and there are no clinical relapse symptoms or imaging progress, it is considered that the MOG-IgG serum titer has turned negative. The antibody status of MOG-IgG serum titer is mainly based on the last follow-up status. Cerebrospinal fluid related examinations include protein content (g/L) and white blood cell count.

### MRI acquisition and processing

Perform optic nerve, brain, and spinal cord MRI scans on pediatric patients using a 1.5 T or 3.0 T Siemens system MRI. Collect T1 weighted series, T2 weighted series, and fluid attenuated inversion recovery (Flair) sequences, with partial sequence signal enhancement, and record the location of the patient's lesion.

### Statistical analyses

All data analysis and statistical chart creation were completed using SPSS 26.0 and Graph Pad Prism 9.0. Continuous variables that follow a normal distribution are represented by mean ± standard deviation, while continuous variables that do not follow a normal distribution are represented by P_50_ (P_25_ ∼ P_75_). Use chi square or Fisher's exact test (for categorical variables) and Mann Whitney U test (for continuous variables) for inter group feature comparison. Use Kaplan Meier method to plot the time of initial conversion to serum MOG-IgG titers. Statistically significant variables were included in multivariate logistic regression. Statistically, *P* < 0.05 indicates a statistically significant difference.

## Results

### Study population

**Figure 1:**
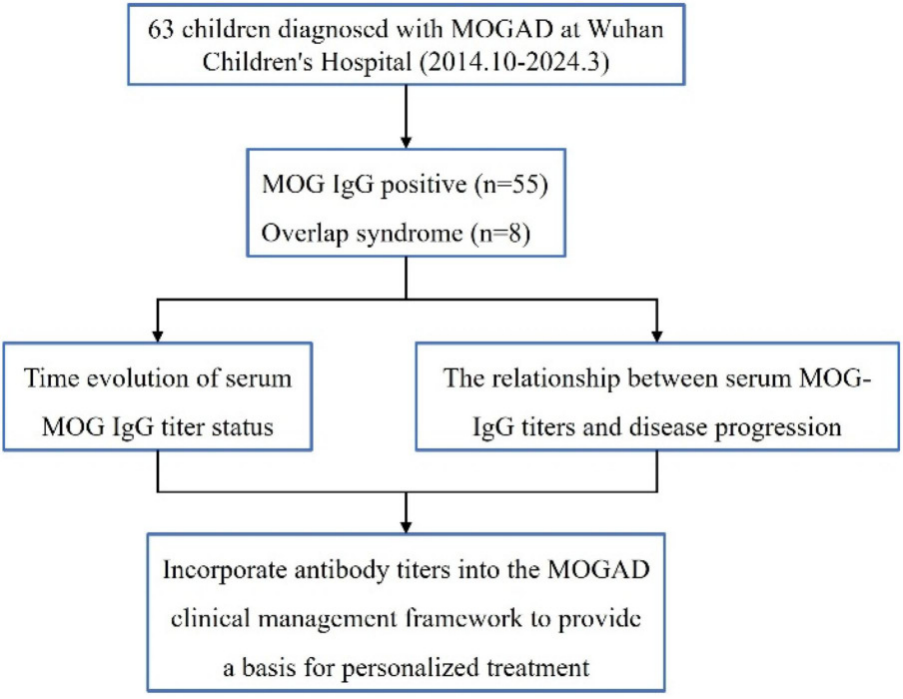
Study flow chart.

This study included a total of 63 children, of whom 55 had positive MOG-IgG serum titers at the onset of the disease, 5 children tested positive for NMDAR-IgG and MOG-IgG, and 3 children tested positive for GFAP-IgG and MOG-IgG. The study flowchart is shown in Figure 1.

The baseline data of 55 children with MOG-IgG positive at onset are shown in Table 1. There were 27 males (49.1%), 28 females (50.9%), and 18 patients (32.7%) with ADEM type clinical presentation at onset. The median MOG-IgG titer at onset was 1:32, and the median time to negative conversion was 3 months. During the follow-up process, 40 patients had monophasic course and 15 patients had relapsing disease course. The median total number of disease courses was 2, the median number of relapses within 3 years was 1, and the median time to initial relapse was 8 months.

**Table 1:** Baseline characteristics of 55 children with MOG-IgG positivity.

Variables	Value
Age at onset, m	91.38 ± 6.38
Gender, No. (%)	
Male	27 (49.1%)
Female	28 (50.9%)
Phenotype at initial onset, No. (%)	
ADEM	18 (32.7%)
Non-ADEM	37 (67.3%)
Disease course nature, No. (%)	
Monophasic course	40 (72.7%)
Relapsing course	15 (27.3%)
Number of disease episodes	2 (2, 3)
Number of relapses within 3 years, No. (%)	
0	1 (6.7%)
1	12 (80.0%)
3	2 (13.3%)
Time to first relapse, m	8 (3, 20)
Preceding infection at initial visit, No. (%)	
Respiratory tract infection	31 (56.4%)
Gastrointestinal tract infection	2 (3.6%)
Respiratory and gastrointestinal tract infection	5 (9.1%)
No infection	17 (30.9%)
Initial MOG-IgG titer at onset	1:32 (1:10, 1:100)
Time to MOG-IgG seroconversion, m	3.00 (1.00, 8.25)
CSF cell count,/*μ*L	42.50 (16.75, 98.50)
CSF protein, g/L	0.40 ± 0.04
Imaging findings at initial visit^a^, No. (%)	
Cortex	23 (41.8%)
Subcortical white matter	25 (45.5%)
Deep white matter	14 (25.5%)
Basal ganglia	14 (25.5%)
Thalamus	16 (29.1%)
Corpus callosum	1 (1.8%)
Brainstem	12 (21.8%)
Cerebellum	17 (30.9%)
Spinal cord	18 (32.7%)
Optic nerve	13 (23.6%)
Treatment at initial visit, No. (%)	
Glucocorticoids	52 (94.5%)
Duration of use, m	6 (6, 6)
Intravenous immunoglobulin	41 (74.5%)
EDSS score at 3-year follow-up	0 (0, 1)
Duration of follow-up, m	68.14 ± 3.81

^a^ Imaging examinations were not performed in 2 children at initial visit.

**Figure 2:**
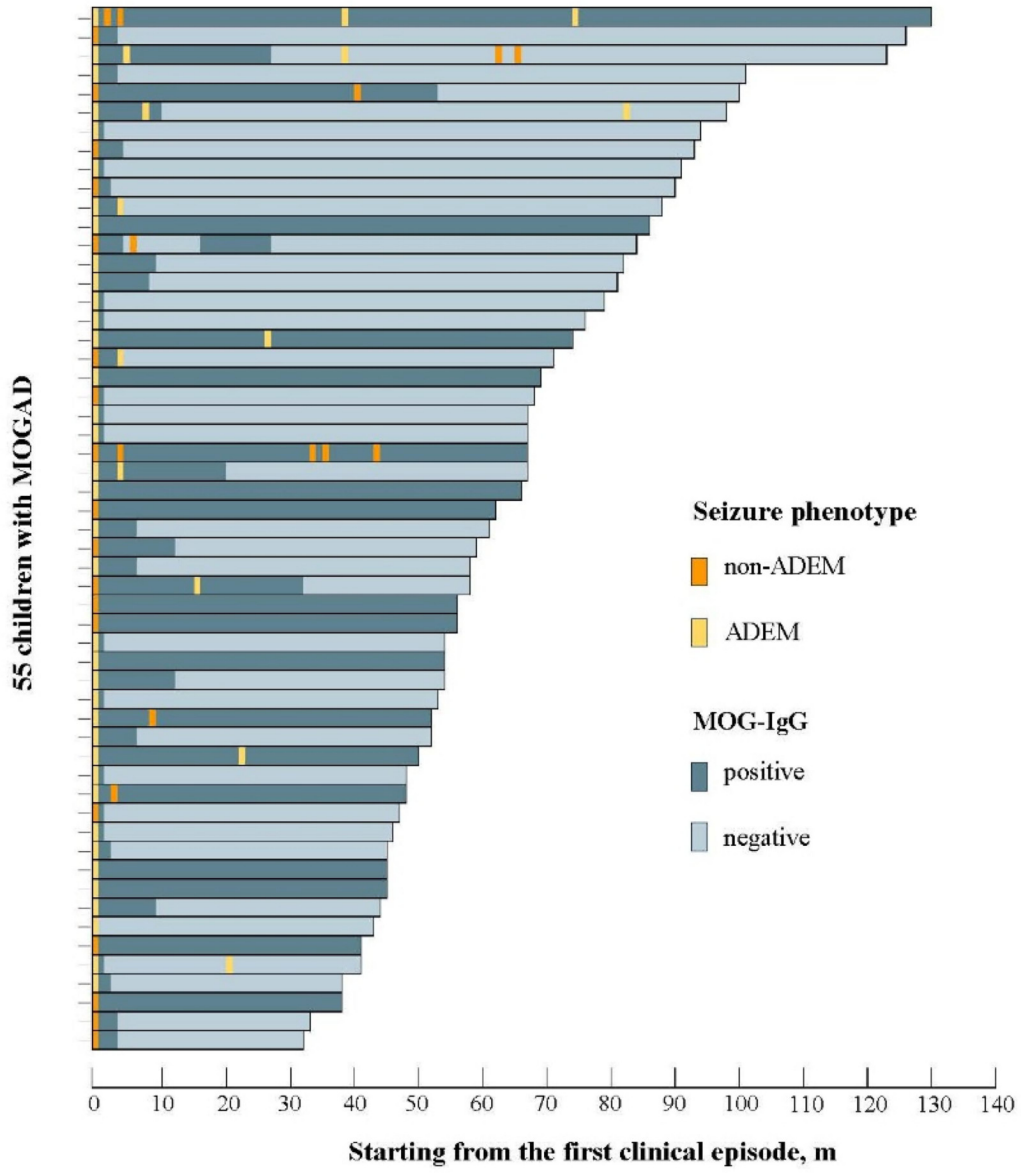
Serological status of 55 participants with positive anti MOG antibodies over time. Each bar represents a participant, with dark blue bars indicating serum positive status and light blue bars indicating serum negative status. Colored squares represent clinical recurrence, with orange squares indicating non ADEM clinical recurrence phenotype and yellow squares indicating ADEM clinical recurrence phenotype.

Figure 2 shows the titer bar chart of 55 MOG-IgG positive children at the onset of the disease during the follow-up process in this study. Among the 15 participants who were MOG-IgG positive with relapsing disease stages, 5 (33.3%) remained serum positive, 9 (60.0%) turned serum negative, and 1 (6.7%) child recovered after serum turned negative. Among 40 children with monophasic disease course, 11 cases (33.3%) remained serum positive and 29 cases (72.5%) turned serum negative.

**Figure 3:**
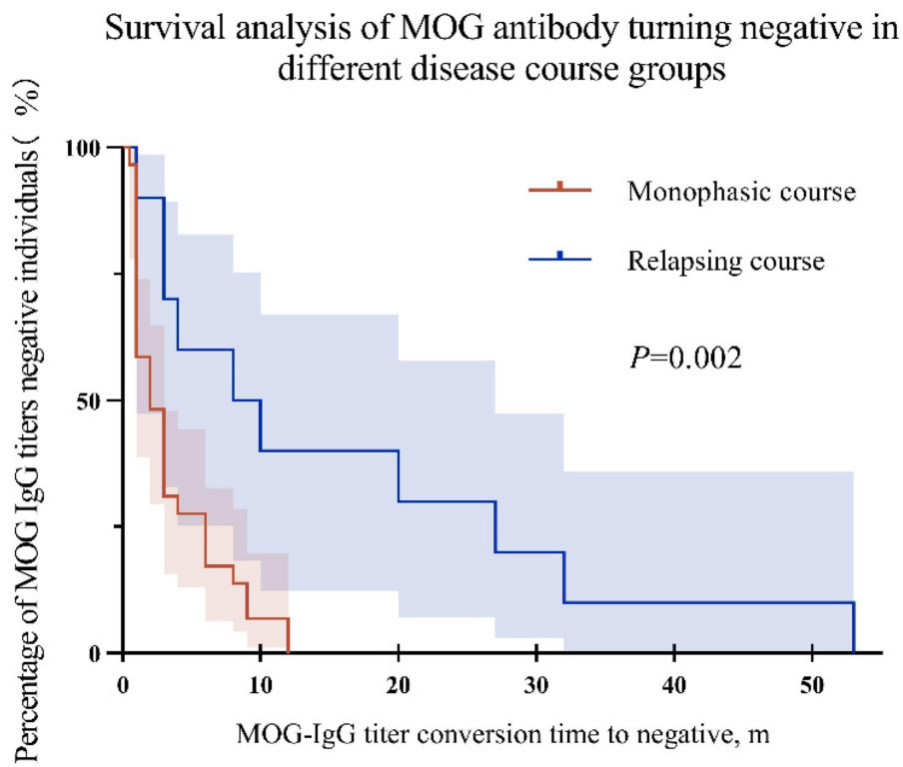
Kaplan Meier curves for monophasic and relapsing disease progression. The shaded area represents 95% CI.

To further investigate the relationship between titers and disease duration, we plotted Kaplan Meier curves of titers turning negative and clinical disease duration in 38 children with MOG-IgG titers turning negative (Figure 3). The results showed that the median time for monophasic disease to turn negative was 2 months, while the median time for relapsing disease to turn negative was 9 months. Compared with children with relapsing disease course, children with single-phase disease course have a shorter time for MOG-IgG to turn negative (*P* = 0.002).

Therefore, we speculate that there is a certain relationship between changes in MOG-IgG serum titers in children and disease outcomes. We further divided the 55 children with MOG-IgG initial titer positive in this study into groups.

### MOG-IgG serum titer status and clinical characteristics in children with MOGAD

In this study, the median time to serum seronegativity among 55 children with MOGAD was 3 months (Table 1). Persistent MOG-IgG was defined as failure to achieve seronegativity within 3 months, while transient positive MOG-IgG was defined as conversion to seronegativity within 3 months or less. Table 2 summarizes the MOG-IgG titer status and clinical characteristics of 55 pediatric patients. Among them, 24 children's MOG-IgG turned negative within 3 months, and 31 children's MOG-IgG remained positive during follow-up for more than 3 months. 21 cases (87.5%) of children with transient positive MOG-IgG titers had monophasic disease course, while 12 cases (38.7%) of children with persistent positive MOG-IgG titers had relapsing disease course. The number of disease courses in the transient positive MOG-IgG titers group was 1.10 ± 0.07, and the number of disease courses in the persistent positive MOG-IgG titers group was 1.56 ± 0.202. Therefore, children with persistent positive MOG-IgG titers have a higher likelihood of relapse compared to those with transient positive MOG-IgG titers (*χ* 2 = 4.685, *P* = 0.03), and the frequency of relapse may be higher (Z = 2.254, *P* = 0.024); The EDSS score for children with persistent positive MOG-IgG titers during the third year follow-up was 0.74 ± 0.16, while the score for children with transient positive MOG-IgG titers was 0.25 ± 0.10. Children with persistent positive MOG-IgG titers may have poor long-term prognosis (Z = 2.490, *P* = 0.013). Variables with statistical significance were included in the multivariate logistic regression analysis. The results indicated that the EDSS score assessed at the 3rd year (prognostic score) was an independent influencing factor for MOG-IgG titer status (positive/negative) (OR: 3.021, 95% CI = 1.030∼8.864, *P* = 0.044). Specifically, children with long-term positive MOG-IgG titers had higher EDSS scores at the 3rd year of follow-up (Table 3).

**Table 2:** Clinical characteristics of pediatric patients stratified by MOG antibody titer Status.

Variables	Transient MOG-IgG Positivity Group (*n* = 24)	Persistent MOG-IgG Positivity Group (*n* = 31)	*χ*2/Z	*P Value*
Age at onset, m	79.5 (52.25, 124.25)	89.00 (64.00, 124.00)	0.645	0.519
Gender, No. (%)			0.439	0.508
Male	13 (54.2%)	14 (45.2%)		
Female	11 (45.8%)	17 (54.8%)		
Phenotype at initial onset, No. (%)			0.245	0.620
ADEM	7 (29.2%)	11 (35.5%)		
Non-ADEM	17 (70.8%)	20 (64.5%)		
Disease course nature, No. (%)			4.685	0.030
Monophasic course	21 (87.5%)	19 (61.3%)		
Relapsing course	3 (12.5%)	12 (38.7%)		
Number of disease episodes	1.10 ± 0.07	1.56 ± 0.202	2.254	0.024
Number of relapses within 3 years^a^, No. (%)			0.296	0.802
0	1 (11.1%)	0		
1	7 (77.8%)	5 (83.3%)		
3	1 (11.1%)	1 (16.7%)		
Time to first relapse, m	15.67 ± 4.206	6.00 ± 2.129	−1.536	0.145
Preceding infection at initial visit, No. (%)			1.686	0.725
Respiratory tract infection	14 (58.3%)	17 (54.8%)		
Gastrointestinal tract infection	0	2 (6.5%)		
Respiratory and gastrointestinal tract infection	2 (8.3%)	3 (9.7%)		
No infection	8 (33.3%)	9 (29.0%)		
CSF cell count,/μL	65.00 ± 15.4	46.00 ± 9.12	−0.283	0.777
CSF protein, g/L	0.41 (0.22, 0.54)	0.30 (0.18, 0.41)	−0.851	0.395
Imaging findings at initial visit, No. (%)				
Cortex	11 (45.8%)	12 (41.4%)	0.106	0.745
Subcortical white matter	10 (41.7%)	15 (51.7%)	0.533	0.465
Deep white matter	5 (20.8%)	9 (31.0%)	0.703	0.402
Basal ganglia	6 (25.0%)	8 (27.6%)	0.045	0.832
Thalamus	6 (25.0%)	10 (34.5%)	0.56	0.454
Corpus callosum	0	1 (3.4%)	0.844	0.358
Brainstem	5 (20.8%)	7 (24.1%)	0.082	0.775
Cerebellum	8 (33.3%)	9 (31.0%)	0.032	0.858
Spinal cord	9 (37.5%)	9 (31.0%)	0.245	0.621
Optic nerve	6 (25.0%)	7 (24.1%)	0.005	0.942
Treatment at initial visit, No. (%)				
Glucocorticoids	22 (91.7%)	30 (96.8%)	0.684	0.408
Duration of use, m	6.00 (6.00, 6.00)	6 (4.98, 9.00)	0.748	0.454
Intravenous immunoglobulin	18 (75.0%)	23 (74.2%)	0.005	0.946
EDSS score at 3-year follow-up	0.25 ± 0.10	0.74 ± 0.16	2.490	0.013
Duration of follow-up, m	60.05 (44.93, 81.18)	60.70 (51.90, 81.80)	0.560	0.575

^a^ Indicates analysis restricted to patients with multiphasic disease course.

ADEM, acute disseminated encephalomyelitis; CSF, cerebrospinal fluid; EDSS, expanded disability status scale; MOG, myelin oligodendrocyte glycoprotein.

**Table 3:** Univariate and multivariate ordinal logistic regression analyses of MOG-IgG serum titer status in MOGAD patients.

Features	*β*	OR (95% CI)	*P* value	*β*	OR (95% CI)	*P* value
Disease course nature（Ref. Monophasic course）	1.486	4.421 (1.080, 18.093）	0.039	1.015	2.758（0.609, 12.490）	0.188
Number of disease episodes	1.209	3.351（0.903, 12.434）	0.071			
EDSS score at 3-year follow-up	1.219	3.384（1.260, 9.086）	0.016	1.106	3.021（1.030, 8.864）	0.044

*β*, coefficient; OR, odds ratio; CI, confidence interval.

### The relationship between clinical characteristics and onset MOG-IgG titers in children with MOGAD

In this study, the median serum titer of 55 children with MOGAD was 1:32 (Table 1). The low MOG-IgG titer group was defined as ≤1:32, and the high MOG-IgG titer group was defined as >1:32. Table 4 summarizes the different MOG-IgG titer groups and clinical characteristics at the onset of the disease. Among them, 36 cases had onset MOG-IgG titers ≤ 1:32, and 19 cases had MOG-IgG titers > 1:32. Among the 50 children who underwent cerebrospinal fluid puncture, the number of cerebrospinal fluid cells in the MOG-IgG high titer group was 48.00 (25.50, 143.00), which was higher than that in the MOG-IgG low titer group at the onset of the disease (Z = 2.100, *P* = 0.036). The duration of glucocorticoids use in the high titer MOG-IgG group is 6.00 (6.00, 12.00) months, while in the low titer MOG-IgG group it is 6.00 (3.00, 6.00) months. One child used glucocorticoids in the early stage, but the duration of glucocorticoids was not clearly recorded, so the statistical analysis of hormone use time was not included. Children in the high titer group used glucocorticoids more frequently (Z = 3.088, *P* = 0.002), and 3 children did not receive glucocorticoids treatment due to parental refusal. Variables with statistical significance were included in the multivariate logistic regression analysis. We found that the duration of glucocorticoids use was an independent influencing factor for serum MOG-IgG titer at disease onset (OR: 1.483, 95% CI = 1.105 ∼ 1.991, *P* = 0.009). Specifically, when the serum MOG-IgG titer was high, the duration of glucocorticoid use was longer (Table 5).

**Table 4:** Clinical characteristics of pediatric patients stratified by MOG antibody titer at onset.

Characteristic	Low MOG Antibody Titer Group at Onset (*n* = 36)	High MOG Antibody Titer Group at Onset (*n* = 19)	χ^2^/Z	*P Value*
Age at onset, m	80.50 (55.25, 115.50)	95.00 (66.00, 128.00)	0.823	0.410
Gender, No. (%)			0.567	0.452
Male	19 (52.8%)	8 (42.1%)		
Female	17 (47.2%)	11 (57.9%)		
Phenotype at initial onset, No. (%)			0.542	0.462
ADEM	13 (36.1%)	5 (26.3%)		
Non-ADEM	23 (63.9%)	14 (73.7%)		
Disease course nature, No. (%)			0.013	0.908
Monophasic course	26 (72.2%)	14 (73.7%)		
Relapsing course	10 (27.8%)	5 (26.3%)		
Number of disease episodes	1.00 (1.00, 2.00)	1.00 (1.00, 2.00)	−0.148	0.883
Number of relapses within 3 years^a^, No. (%)			0.364	0.903
0	1 (9.1%)	0		
1	8 (72.7%)	4 (100%)		
3	2 (18.2%)	0		
Time to first relapse, m	13.91 ± 3.627	6.00 ± 3.109	−1.309	0.226
Preceding infection history, No. (%)			4.784	0.192
Respiratory tract infection	24 (66.7%)	8 (42.1%)		
Gastrointestinal tract infection	1 (2.8%)	7 (36.8%)		
Respiratory and gastrointestinal tract infection	2 (5.6%)	1 (5.3%)		
No infection	9 (25.0%)	8 (42.1%)		
CSF cell count^a^,/μL	22.00 (12.00, 58.00)	48.00 (25.50, 143.00)	2.100	0.036
CSF protein^a^, g/L	0.28 (0.19, 0.44)	0.36 (0.29, 0.53)	1.711	0.087
Treatment at initial visit				
Glucocorticoids	34 (94.4%)	18 (94.7%)	0.002	1.000
Duration of use^a^, m	6.00 (3.00, 6.00)	6.00 (6.00, 12.00)	3.088	0.002
Intravenous immunoglobulin	26 (72.2%)	15 (78.9%)	0.296	0.749
EDSS score at 3-year follow-up	1.00 (0.00, 1.30)	1.00 (0.00, 3.00)	0.795	0.427

^a^ Low titer is defined as antibody titer <1:100, and high titer as ≥ 1:100; Cerebrospinal fluid (CSF) examination was not performed in 5 patients at initial visit, and 4 patients did not receive corticosteroid treatment at initial visit; Number of relapses within 3 years was analyzed in patients with multiphasic disease course.

**Table 5:** Univariate and multivariate ordinal logistic regression analyses of MOG-IgG titer at onset in MOGAD patients.

Features	*β*	OR (95% CI)	*P* value	*β*	OR (95% CI)	*P* value
CSF cell count	0.008	1.008 (0.999, 1.017)	0.084			
Duration of glucocorticoids use	0.412	1.510 (1.131, 2.017)	0.005	0.394	1.483 (1.105, 1.991)	0.009

*β*. coefficient; OR, odds ratio; CI, confidence interval.

### Time evolution of MOG-IgG serum titer status

**Figure 4:**
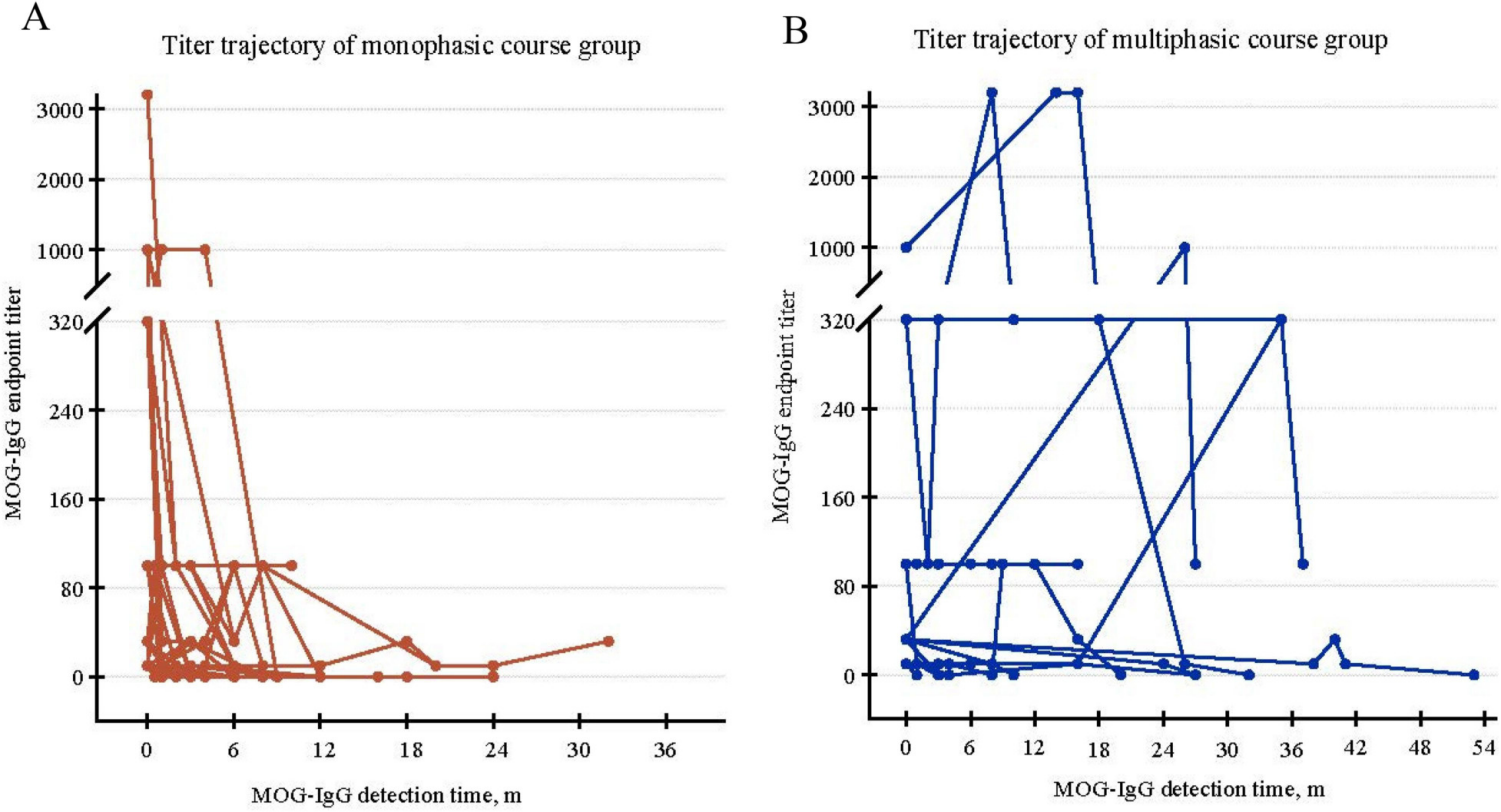
Continuous MOG titer trajectory of children with positive anti MOG antibodies (**(A)** monophasic disease group, **(B)** relapsing disease group). The data points represent the titers measured in each serological assessment.

Figure 4 shows the continuous MOG-IgG titer trajectory of MOG-IgG positive children in different disease course groups. The titer trajectory of 40 children with monophasic disease course showed a trend of MOG-IgG titer gradually changing from high titer to low titer until it turned negative. However, among 15 children with relapsing course, the MOG-IgG titer showed different manifestations, mainly manifested as an initial increase and then decrease in MOG-IgG titer, followed by a persistent positive state or a negative state.

### Characteristics of antibody titer changes in special cases

This study included 8 children with overlap syndrome. The clinical baseline data are shown in Table 6, including 5 children with MOG-IgG and NMDAR-IgG positivity, and 3 children with MOG-IgG and GFAP-IgG positivity. In this study, the average age of children with overlap syndrome was 71.29 ± 11.36 months, with 3 cases (37.5%) showing monophasic disease course and 5 cases (62.5%) showing relapsing recurrent disease course. One patient's MOG-IgG titer remained positive during follow-up, and the median time for MOG-IgG titers to turn negative in seven patients was 6 months.

**Table 6:** Baseline characteristics of 8 pediatric patients with overlap syndrome.

Characteristic	Value
Age at onset, m	71.29 ± 11.36
Gender, No. (%)	
Male	6 (75.0%)
Female	2 (25.0%)
Overlap syndrome antibody type^a^	
Anti-NMDAR antibody IgG	5 (62.5%)
Anti-GFAP antibody IgG	3 (37.5%)
Phenotype at initial onset, No. (%)	
ADEM	4 (50.0%)
Non-ADEM	4 (50.0%)
Disease course nature, No. (%)	
Monophasic course	3 (37.5%)
Relapsing course	5 (62.5%)
Number of disease episodes	2 (1, 3)
Number of relapses within 3 years, No. (%)	
1	2 (40.0%)
2	3 (60.0%)
Time to first relapse, m	12.00 (4.50, 14.50)
Preceding infection at initial visit, No. (%)	
Respiratory tract infection	2 (25.0%)
Gastrointestinal tract infection	3 (37.5%)
Respiratory and gastrointestinal tract infection	1 (12.5%)
No infection	2 (25.0%)
Initial MOG-IgG titer at onset	1:10 (1:10, 1:32)
Time to MOG-IgG seroconversion, m	6 (3, 16)
CSF cell count,/μL	33 (9, 65)
CSF protein, g/L	0.45 ± 0.10
Imaging findings at initial visit, No. (%)	
Cortex	6 (75.0%)
Subcortical white matter	5 (62.5%)
Deep white matter	1 (12.5%)
Basal ganglia	2 (25.0%)
Thalamus	2 (25.0%)
Corpus callosum	0
Brainstem	0
Cerebellum	0
Spinal cord	0
Optic nerve	2 (25.0%)
Treatment at initial visit, No. (%)	
Glucocorticoids	8 (100%)
Duration of use, m	6.48 (6.00, 7.20)
Intravenous immunoglobulin	6 (75.0%)
EDSS score at 3-year follow-up	0 (0, 2)
Duration of follow-up, m	36.50 (36.00, 41.50)

^a^ All patients were positive for anti-MOG IgG.

## Discussion

Myelin oligodendrocyte glycoprotein antibody-associated disease (MOGAD), as an autoimmune disease of the central nervous system, exhibits marked heterogeneity in its clinical course (13). Previous studies have reported that 11%–47% of patients may experience a relapse within 2 years (14).The relapse rate in children is significantly higher than that in adults, with 46% of pediatric patients experiencing relapse within an average follow-up period of 3.9 years (15). About 27.3% of the patients in this study showed relapsing disease course, while the remaining patients showed monophasic disease course during the follow-up period. In this disease spectrum, the dynamic changes in antibody titers have gradually become a key biological indicator for predicting disease behavior and outcomes.

Some studies have shown that higher MOG-IgG titers are associated with an increased risk of relapse. For example, studies have found that serum MOG-IgG titers ≥ 1:100 or ≥ 1:32 are independent risk factors for relapse (7), consistent with this study. Similarly, persistent positivity of MOG-IgG is also considered a high-risk factor for relapse. Children with persistent antibody positivity (titer ≥ 1:160 or fluctuating after 6 months of treatment) have a significantly increased risk of relapse (63% −75%), while those who turn negative early have a relapse rate of <20% (16). A decrease or negative antibody titer is significantly associated with a reduced risk of relapse (17). However, some studies have put forward different viewpoints. A study on children found that the acute phase MOG-IgG titers did not increase in relapsing patients and should not be the sole basis for predicting relapse and guiding long-term treatment (11). Another study also suggests that the MOG-IgG titers at the onset of the disease are not related to specific disease progression (10). These controversies may stem from differences in research design, patient populations, testing methods, and titer cutoff values. In addition, there is also discussion about the significance of MOG-IgG in cerebrospinal fluid. A study has found that positive MOG-IgG in cerebrospinal fluid, especially in intrathecal synthesis, is associated with more severe clinical manifestations and poorer prognosis (18). This study only explored the relationship between serum MOG-IgG and prognosis. Therefore, larger prospective studies are needed in the future to unify detection standards, clarify the predictive value of MOG-IgG titers and their dynamic changes in different clinical subtypes and populations, and resolve current controversies.

However, the reported risk factors for the relapse of MOGAD in children are still unclear and controversial. Therefore, in this study, we focused on exploring the risk factors and antibody titer characteristics of MOGAD relapse in children. The data from this queue shows that children with persistent positive MOG-IgG titers have a significantly higher likelihood of relapse than those with transient positive titers (*P* < 0.05), the time for MOG-IgG antibody to turn negative in children with monophasic disease course was significantly shorter than that in children with relapsing disease course (*P* < 0.05). A multicenter study in Italy confirmed that MOG-IgG negative conversion can reduce the risk of relapse by 95% (IRR=0.05, *P* < 0.001) (17). In patients with MOGAD, an initial remission MOG-IgG titer >1:2560 is an independent predictor of future relapse, with a 6.9-fold higher relapse risk compared to patients with low titers (HR 6.87, 95% CI 2.44–19.41) (17). This may have important clinical relevance because seronegative pediatric patients have been shown to have a significantly lower risk of relapse (19, 20). These results provide a rationale for supporting persistent antibody positivity as a biomarker for poor prognosis. In addition, complement protein (21), cytokines (22), and neurofilament light chains (23) have also been studied as biomarkers in determining the prognosis of MOGAD.

Not all children with persistent positive serum MOG-IgG titers will experience relapse. We found that among the 15 MOG-IgG positive participants with multiphase disease, 5 (33.3%) were persistent serum positive, 1 (6.7%) had fluctuating serum status, and 9 (60.0%) turned serum negative (Figure 3). Among 40 children with monophasic disease course, 11 cases (27.5%) remained serum positive and 29 cases (72.5%) turned serum negative. Persistent seropositivity on serial MOG-IgG measurement has been significantly associated with relapsing disease course (24, 25); however, the literature lacks consensus regarding this relationship (17, 26). Conversion to MOG-IgG seronegative status appears to confer a reduced risk of subsequent relapse (17, 26). Some patients may experience antibody fluctuations, from positive to negative and back to positive, which makes it more difficult to predict disease relapse based on antibody titers.

How to predict the relapse of MOGAD earlier and more accurately is the key to evaluating prognosis and early intervention. Exploring the risk factors for relapse after onset in order to initiate immunotherapy in a timely manner to prevent relapse may help address this issue, which is currently a hot research topic. Research has found that long-term immunosuppressive maintenance therapy (mainly including azathioprine, mycophenolate mofetil, rituximab, and regular IVIG) may reduce the frequency of relapse (27). After the first episode, glucocorticoids maintenance for an appropriate period following discharge is important for achieving a favorable long-term prognosis, particularly in patients with a high EDSS score at discharge and those at a heightened risk of relapse (14). In a study of adult MOGAD patients in China, optic neuritis was the most common clinical phenotype (54.8%). The study also found that the presence of visual symptoms at onset was an independent risk factor for long-term disability (EDSS ≥ 3) (OR 10.49) (28). The number of cerebrospinal fluid cells in the MOG-IgG high titer group was significantly higher than that in the low titer group (*P* < 0.05). A queue study conducted in South Korea in 2025 found that the median number of nucleated cells in cerebrospinal fluid of the high titer group (titer ≥ 1:100) was 98*10 ⁶/L, significantly higher than the 32*10⁶/L of the low titer group (titer < 1:100) (*P* < 0.01). The correlation between antibody titer and cerebrospinal fluid cell count is high (29). MOGAD is an autoantibody-mediated pathology that arises due to the cooperation of MOG-specific B- and T-cells (30). High MOG-IgG titers indicate peripheral B cell clonal expansion and migration to the central nervous system (CNS). Therefore, the dosage and timing of immunotherapy are very important. Clinical observations have found that the optimal dose of 12.5 mg prednisone per day for adults (0.16 mg/kg/day for children) can delay the onset of MOGAD for at least 3 months and delay the time of first relapse (31). The relapse of high titer patients during standard glucocorticoids reduction suggests that these patients may require more aggressive immunosuppressive therapy, which is consistent with the results of this study. However, this may also be related to the longer use of glucocorticoids when the patient's clinical symptoms are more severe, and subsequent studies can control for confounding factors to further explore the relationship between treatment and MOG-IgG titer. The initial sample size limited the power for more robust multivariate analysis, but our findings provide important preliminary hypotheses and data support for future research.

Indeed, the proportion of ADEM phenotype in our study was 32.7%, slightly lower than that reported in some countries. This may be attributed to the fact that we strictly applied diagnostic criteria to clearly distinguish ADEM from optic neuritis, myelitis and other phenotypes, and that clinical phenotypes of MOGAD may differ between East Asian and Western populations (32, 33). Further multicenter studies across different ethnicities are warranted in the future. However, due to the small sample size, we only describe the clinical features of overlap syndromes. The acute phase symptoms of MOG-NMDAR overlap syndrome (MNOS) in children, such as seizures and behavioral abnormalities, require a longer period of relief, with a median clinical relief time of 6–8 weeks. Although first-line immunotherapy is sensitive, the relapse rate during the reduction period is as high as 72.7% (8/11 cases) (34). GFAP-IgG mainly targets astrocytes, inducing meningitis or myelitis. The pathology is mainly characterized by acute inflammatory infiltration, with less formation of chronic B cell nests. The two work together to form a dual damage of neurons and glial cells, promoting the survival of central B cells and long-term production of antibodies (35). Therefore, in clinical practice, second-line immunosuppressants should be used early in children with overlap syndrome, and the clearance of relevant immune antibodies should be dynamically monitored to guide the course of treatment.

The current research has some limitations. Firstly, the single center design resulted in limited sample size, as we analyzed a cohort of pediatric patients in which the majority of serum samples were collected during acute exacerbations. However, there were still a very small number of patients who underwent MOG-IgG testing one year after onset. Secondly, the clinical, MRI, and laboratory features of MOGAD seem to be relatively common in studies of different geographic regions and ethnicities, but our results in the Chinese pediatric patient cohort may need to be confirmed in other ethnicities. Thirdly, this study only enrolled children with serum MOG-IgG positivity, as we focused on the prognostic value of serum MOG-IgG titers. Lumbar puncture is an invasive procedure in children, and some families declined the examination, therefore, MOG-IgG in cerebrospinal fluid was not further analyzed.

In summary, this study revealed the correlation between the persistent status, titer level, and rate of seroconversion of MOG-IgG and the risk of disease relapse, functional prognosis, and treatment response through longitudinal monitoring of antibody titers in children with MOGAD, providing objective evidence for clinical management.

## Conclusion

For children with high MOG-IgG titers at onset or those who have not turned negative within 3 months, extending the immunotherapy cycle may be considered; The persistent positive status of MOG-IgG is associated with relapse and poor prognosis; The time for MOG-IgG titer to turn negative is related to the course of the disease: children with single-phase disease have a shorter time for MOG-IgG titer to turn negative than those with multi-phase disease. MOG-IgG titer levels affect inflammatory activity and treatment needs: the number of cerebrospinal fluid cells in the high titer group increased significantly, as longer glucocorticoid therapy cycles could be considered. There is heterogeneity in the dynamic spectrum of MOG-IgG titers. The above findings support the inclusion of dynamic monitoring of MOG-IgG titers in the clinical management framework of pediatric MOGAD, providing a basis for personalized treatment.

## Data Availability

The raw data supporting the conclusions of this article will be made available by the authors, without undue reservation.

## References

[1] Takai Y Misu T Fujihara K Aoki M. Pathology of myelin oligodendrocyte glycoprotein antibody-associated disease: a comparison with multiple sclerosis and aquaporin 4 antibody-positive neuromyelitis optica spectrum disorders. Front Neurol. (2023) 14:1209749. 10.3389/fneur.2023.1209749 37545724 PMC10400774

[2] Deschamps R Guillaume J Ciron J Audoin B Ruet A Maillart E Early maintenance treatment initiation and relapse risk mitigation after a first event of MOGAD in adults: the MOGADOR2 study. Neurology. (2024) 103(3):e209624. 10.1212/WNL.0000000000209624 38991174

[3] Du Y Xiao L Ding Z Huang K Xiao B Feng L. MOGAD involving cranial neuropathies: a case report and review of literature. Brain Sci. (2022) 12(11):1529. 10.3390/brainsci12111529 36421853 PMC9688642

[4] Boudjani H Fadda G Dufort G Antel J Giacomini P Levesque-Roy M Clinical course, imaging, and pathological features of 45 adult and pediatric cases of myelin oligodendrocyte glycoprotein antibody-associated disease. Mult Scler Relat Disord. (2023) 76:104787. 10.1016/j.msard.2023.104787 37320939

[5] Cheng J Wang Z Wang J Pang X Wang J Zhang M The nomogram model predicts relapse risk in myelin oligodendrocyte glycoprotein antibody-associated disease: a single-center study. Front Immunol. (2025) 16:1527057. 10.3389/fimmu.2025.1527057 40098969 PMC11911489

[6] Martin K Srikanth P Kanwar A Falardeau J Pettersson D Yadav V. Clinical and radiographic features of a cohort of adult and pediatric subjects in the Pacific northwest with myelin oligodendrocyte glycoprotein antibody-associated disease (MOGAD). Mult Scler Relat Disord. (2024) 81:105130. 10.1016/j.msard.2023.105130 37979410 PMC10842716

[7] Wang J Yang K Zhang F Yi Y Wang J. Clinical risk factors for recurrence of myelin oligodendrocyte glycoprotein antibody-associated disease. Mult Scler Relat Disord. (2023) 77:104879. 10.1016/j.msard.2023.104879 37442076

[8] Molazadeh N Bilodeau PA Salky R Bose G Lotan I Romanow G Predictors of relapsing disease course following index event in myelin oligodendrocyte glycoprotein antibody-associated disease (MOGAD). J Neurol Sci. (2024) 458:122909. 10.1016/j.jns.2024.122909 38335710

[9] Roy S Vasileiou E Barreras P Ahmadi G Chen H Suslovic W Longitudinal evaluation of serum MOG-IgG titers in MOGAD after initiation of maintenance immunoglobulin: a case series. Mult Scler. (2024) 30(4–5):594–9. 10.1177/13524585231211119 38018493

[10] Brown AM Sridhar A Gliksman F Thomas FP Pandey KS. Clinical characteristics of pediatric and adult myelin oligodendrocyte antibody-associated disease (MOGAD): a single-center study in the northeast. Mult Scler Relat Disord. (2024) 92:105950. 10.1016/j.msard.2024.105950 39541821

[11] Wang X Zhao R Yang H Liu C Wang W Liu T Clinical analysis of myelin oligodendrocyte glycoprotein antibody-associated demyelination in children: a single-center cohort study in China. Mult Scler Relat Disord. (2022) 58:103526. 10.1016/j.msard.2022.103526 35063909

[12] ZhangBao J Huang W Zhou L Tan H Wang L Wang M Clinical feature and disease outcome in patients with myelin oligodendrocyte glycoprotein antibody-associated disorder: a Chinese study. J Neurol Neurosurg Psychiatry. (2023) 94(10):825–34. 10.1136/jnnp-2022-330901 37321840

[13] Cobo-Calvo A Ruiz A Maillart E Audoin B Zephir H Bourre B Clinical spectrum and prognostic value of CNS MOG autoimmunity in adults: the MOGADOR study. Neurology. (2018) 90(21):e1858–69. 10.1212/WNL.0000000000005560 29695592

[14] Sun W Xie Y Han A Zhou X Zhang S Xie Y Clinical characteristics and factors associated with recurrence and long-term prognosis in patients with MOGAD. Front Immunol. (2025) 16:1535571. 10.3389/fimmu.2025.1535571 40406135 PMC12095161

[15] Virupakshaiah A Schoeps VA Race J Waltz M Sharayah S Nasr Z Predictors of a relapsing course in myelin oligodendrocyte glycoprotein antibody-associated disease. J Neurol Neurosurg Psychiatry. (2025) 96(1):68–75. 10.1136/jnnp-2024-333464 PMC11652255 38964848

[16] Tisavipat N Wilf-Yarkoni A Al-Ani A Costello F Kosiyakul P Jitprapaikulsan J Relapse and disability outcomes in incident MOGAD patients undergoing watchful waiting after onset. Mult Scler Relat Disord. (2025) 102:106631. 10.1016/j.msard.2025.106631 40712508 PMC12318300

[17] Gastaldi M Foiadelli T Greco G Scaranzin S Rigoni E Masciocchi S Prognostic relevance of quantitative and longitudinal MOG antibody testing in patients with MOGAD: a multicentre retrospective study. J Neurol Neurosurg Psychiatry. (2023) 94(3):201–10. 10.1136/jnnp-2022-330237 36460438

[18] Carta S Cobo CÁ Armangué T Saiz A Lechner C Rostásy K Significance of myelin oligodendrocyte glycoprotein antibodies in CSF: a retrospective multicenter study. Neurology. (2023) 100(11):e1095–108. 10.1212/WNL.0000000000201662 36526426 PMC10074465

[19] Waters P Fadda G Woodhall M O’Mahony J Brown RA Castro DA Serial anti-myelin oligodendrocyte glycoprotein antibody analyses and outcomes in children with demyelinating syndromes. JAMA Neurol. (2020) 77(1):82–93. 10.1001/jamaneurol.2019.2940 31545352 PMC6763982

[20] Armangue T Olivé-Cirera G Martínez-Hernandez E Sepulveda M Ruiz-Garcia R Muñoz-Batista M Associations of paediatric demyelinating and encephalitic syndromes with myelin oligodendrocyte glycoprotein antibodies: a multicentre observational study. Lancet Neurol. (2020) 19(3):234–46. 10.1016/S1474-4422(19)30488-0 32057303

[21] Keller CW Lopez JA Wendel EM Ramanathan S Gross CC Klotz L Complement activation is a prominent feature of MOGAD. Ann Neurol. (2021) 90(6):976–82. 10.1002/ana.26226 34569094

[22] Arru G Sechi E Mariotto S Zarbo IR Ferrari S Gajofatto A Antibody response against HERV-W in patients with MOG-IgG associated disorders, multiple sclerosis and NMOSD. J Neuroimmunol. (2020) 338:577110. 10.1016/j.jneuroim.2019.577110 31715457

[23] Kim H Lee EJ Kim S Choi LK Kim K Kim HW Serum biomarkers in myelin oligodendrocyte glycoprotein antibody-associated disease. Neurol Neuroimmunol Neuroinflamm. (2020) 7(3):e708. 10.1212/NXI.0000000000000708 32184342 PMC7136043

[24] López-Chiriboga AS Majed M Fryer J Dubey D McKeon A Flanagan EP Association of MOG-IgG serostatus with relapse after acute disseminated encephalomyelitis and proposed diagnostic criteria for MOG-IgG-associated disorders. JAMA Neurol. (2018) 75(11):1355–63. 10.1001/jamaneurol.2018.1814 30014148 PMC6248120

[25] Hennes EM Baumann M Schanda K Anlar B Bajer-Kornek B Blaschek A Prognostic relevance of MOG antibodies in children with an acquired demyelinating syndrome. Neurology. (2017) 89(9):900–8. 10.1212/WNL.0000000000004312 28768844

[26] Huda S Whittam D Jackson R Karthikeayan V Kelly P Linaker S Predictors of relapse in MOG antibody associated disease: a cohort study. BMJ Open. (2021) 11(11):e055392. 10.1136/bmjopen-2021-055392 34848526 PMC8634280

[27] Sechi E Cacciaguerra L Chen JJ Mariotto S Fadda G Dinoto A Myelin oligodendrocyte glycoprotein antibody-associated disease (MOGAD): a review of clinical and MRI features, Diagnosis, and management. Front Neurol. (2022) 13:885218. 10.3389/fneur.2022.885218 35785363 PMC9247462

[28] Dai Y Yin Q Han B Yuan T Su K Dai W MOG-IgG detection in serum and cerebrospinal fluid: diagnostic implications and clinical correlates in adult-onset Chinese MOGAD patients. J Neurol. (2025) 272(8):528. 10.1007/s00415-025-13278-8 40694125

[29] Lee S Kong J Lyu S Nam SO Lim TJ Song JY Relationship between cerebrospinal fluid cytokines/chemokines and clinical impact of myelin oligodendrocyte glycoprotein antibody-associated disorders in children. Brain Dev. (2025) 47(4):104389. 10.1016/j.braindev.2025.104389 40614442

[30] Andersen J Brilot F. Myelin oligodendrocyte glycoprotein antibody-associated disease (MOGAD): insights into pathogenesis and biomarkers of prognosis. Semin Immunol. (2025) 78:101944. 10.1016/j.smim.2025.101944 40088708

[31] Trewin BP Dale RC Qiu J Chu M Jeyakumar N Dela Cruz F Oral corticosteroid dosage and taper duration at onset in myelin oligodendrocyte glycoprotein antibody-associated disease influences time to first relapse. J Neurol Neurosurg Psychiatry. (2024) 95(11):1054–63. 10.1136/jnnp-2024-333463 38744459 PMC11503134

[32] Gklinos P Dobson R Myelin oligodendrocyte glycoprotein-antibody associated disease: an updated review of the clinical spectrum, pathogenetic mechanisms and therapeutic management. Antibodies (Basel). (2024) 13(2):43. 10.3390/antib13020043 38804311 PMC11130828

[33] Hooshmand SJ Vorasoot N Li X Chen JJ Redenbaugh V Banks SA Application of the ADEM definition to cerebral attacks of MOG antibody-associated disease. Mult Scler. (2025) 31(14):1629–40. 10.1177/13524585251359450 40827744 PMC12421408

[34] Gong S Zhang WH Ren HT Li JW Zhou J Cheng H Clinical observation on the overlapping syndrome of myelin oligodendrocyte glycoprotein antibody and anti-N-methyl-D aspartate receptor in children. Zhonghua Er Ke Za Zhi. (2020) 58(7):581–5. 10.3760/cma.j.cn112140-20191209-00788 32605343

[35] Fang T Wu W He X Liang Y Lin Q Dai K Clinical characteristics of overlapping syndrome in patients with GFAP-IgG and MOG-IgG: a case series of 8 patients and literature review. J Neurol. (2024) 271(10):6811–21. 10.1007/s00415-024-12633-5 39190107

